# Tamponade or Filling Effect: Changes of Forces in Myopic Eyes

**DOI:** 10.1155/2014/618382

**Published:** 2014-07-02

**Authors:** Francesco Semeraro, Francesco Morescalchi, Andrea Russo, Mario R. Romano, Ciro Costagliola

**Affiliations:** ^1^Eye Clinic, Department of Neurological and Vision Sciences, University of Brescia, Piazzale Spedali Civili 1, 25123 Brescia, Italy; ^2^Eye Clinic, Istituto Clinico Humanitas, Via Manzoni 56, 20089 Milano, Italy; ^3^Eye Clinic, Department of Health Sciences, University of Molise, Via de Santis, 86100 Campobasso, Italy

## Abstract

Myopia is the most common ocular abnormality. Its high and growing prevalence has contributed to a recent surge in surgical interest in the disorder, since retinal detachment in eyes with high myopia differs from that in emmetropic eyes or eyes with low myopia. The myopic eye, because of its specific anatomy, poses special challenges that need to be overcome to ensure the appropriate use of vitreous substitutes. However, intraocular tamponades have shown great potential for revolutionizing retinal detachment surgery and vitreomacular surgery in general in myopic eyes. We provide an updated review of the clinical use of vitreous substitutes in the myopic eye, paying particular attention to analyzing the ideal function of endotamponade agents and comparing the effects of these agents on the physical and biological properties of the eye.

## 1. Introduction

Pathologic or high myopia (defined as a spherical equivalent greater than −6.00 diopters or an axial length >26.5 mm) [1] is caused by excessive widening and elongation of the eye and is often associated with degenerative changes in the posterior sclera, choroid, Bruch's membrane, retinal pigment epithelium, and neural retina. Moreover, vitreoretinal degenerations such as lattice degeneration, increased rate of vitreous liquefaction, and posterior vitreous detachment are also more common in myopic patients [2].

High myopia often coexists with or contributes to the worsening of other vitreomacular interface disorders that also occur in the nonmyopic population, primarily macular epiretinal membranes (ERM), vitreomacular traction syndrome, macular holes, and macular schisis. Furthermore, the larger volume of the eye increases the shear retinal stress and the traction exerted by the vitreous on the retina during eye movement. Therefore, myopic individuals have a higher incidence and prevalence of retinal detachment (RD). Furthermore, individuals with over 3 diopters of myopia have a 10-fold greater probability of RD relative to the normal population [3, 4].

From a surgical point of view, RD in eyes with high myopia is different from that in emmetropic eyes or eyes with low myopia. Because of the very long axial length and the generalized thinning of the sclera, muscle avulsion, vortex vein damage, hemorrhage, retention of subretinal fluid after internal drainage, and risk of globe perforation have been noted during surgery in highly myopic eyes [5, 6].

Intraocular tamponades have shown potential for revolutionizing RD surgery and vitreomacular surgery in general. The myopic eye, because of its specific anatomy, poses a number of unique challenges that have to be overcome to ensure appropriate use of vitreous substitutes. The presence of a large vitreous cavity, posterior staphyloma, extensive posterior areas of choroidal atrophy, and stronger posterior vitreoretinal adhesions are factors associated with a higher incidence of postoperative complications. It is difficult for any tamponade to be successful in such eyes because of the difficulties associated with complete cortical vitreous separation and the steep edge of the staphyloma relative to the round profile of the tamponade bubble. Indeed, a marked scleral irregularity was found to be associated with the failure of silicone or heavy silicone oil (HSO) to tamponade irregular eye profiles [7].

This review focuses on issues related to the use of vitreous substitutes and tamponades in high myopic eyes, particularly the ideal function of endotamponade agents and the variation in their effects according to the physical and biological properties of the eye.

## 2. Myopia and Vitreoretinal Disease

Although moderate degrees of myopia are relatively common, occurring in approximately 25% of the population in Western countries, pathologic myopia has a much lower prevalence, occurring in approximately 0.3% of the population [8, 9]. Pathologic myopia is ranked as the second to fifth most frequent cause of blindness in studies involving Caucasian populations and is the most frequent cause of blindness in China [10]. Two studies of European populations report that pathologic myopia is the cause of low vision or blindness in 5.8% and 7.8% of eyes with low vision or blindness [11, 12]. These percentages are much higher in Asia, where, in the 3 large studies performed in China and Japan, pathologic myopia was the cause of blindness or low vision in 12.2–27.4% of individuals with low vision [13–15]. The prevalence of high myopia in the European population is estimated to be 0.11–0.47% [11, 12, 16], whereas its prevalence in the Asian population is estimated to be 0.17–1.41% [13–15, 17, 18].

Anatomic and radiographic studies of the shape of the vitreous chamber have revealed some differences between myopic and emmetropic eyes. In emmetropic eyes, the length of the anteroposterior axis is usually slightly shorter than that of the vertical and horizontal axes, and thus the morphology of the vitreous chamber approximates an ellipsoid. In myopic eyes, all three axes are increased in length, particularly the anteroposterior axis. Therefore, myopic eyes are typically both larger and longer and are closer to a spherical shape than emmetropic eyes. In very high myopia, the anteroposterior axis is longer than the other two axes, and the vitreous chamber may be prolate [19, 20].

A shear stress is defined as the component of stress coplanar with a material cross section; it arises from the force vector component parallel to the cross section. A recent theory hypothesized that the enlargement and elongation of the vitreous chamber might increase the vitreous and retinal shear stress exerted by the movement of the eye, contributing to the pathogenesis of posterior vitreous detachment and RD [21]. The shear stress produced by eye movements could be the origin of the disintegration of the collagen network that leads to vitreous liquefaction and posterior vitreous detachment. During physiologic rotations of the eye, the vitreous, especially if already partially liquefied, is more rapidly disrupted in larger eyes than in normal eyes [21]. In pathologic myopia, other mechanical factors promote RD. In particular, a thinner retina is more easily damaged and leads to degenerative changes in the posterior fundus that consequently cause visual dysfunction [22–24].

Abnormal posterior elongation of the globe is associated with thinning of the sclera and posteriorly localized scleral ectasia, which is known as posterior staphyloma. This ectasia is strongly associated with the development of other pathologic sequelae that occur as a result of progressive biomechanical stretching of the ocular coats in the myopic globe [25]. The prevalence of posterior staphyloma in pathologic myopia ranged from 19% in a study with eyes longer than 26.5 mm to 35.4% in a histopathologic study [25, 26]. However, a more recent study in eyes with myopic refraction reported a 76% incidence of posterior staphyloma [2]. Posterior staphylomas commonly involve the posterior pole, spreading across the entire macula and up to 3–5 disc diameters nasal to the optic nerve. In some cases, compound staphylomas can occur. In such cases, multiple primary staphylomas overlap in stepped or terraced areas of ectasia. Staphylomas naturally tend to expand and deepen their contours with age [26].

Progressive elongation of the globe and enlargement of the posterior staphyloma are often accompanied by abnormalities of the vitreoretinal interfaces, and they exert a stretching force on the relatively inelastic retinal and choroidal layers. During eye movements, the stress exerted by the vitreous movement on the posterior pole increases with the diameter of the globe, and it might cause a proliferative cellular reaction leading to the formation of ERMs [21, 27, 28].

A progressive alteration of the structure and content of the scleral collagen has been reported in high myopia. Such changes appear to be secondary to both decreased collagen synthesis and accelerated collagen turnover [29]. In ectatic sclera, collagen type I production is reduced, whereas collagen types III and V are more abundant. This progressive alteration of the collagen, which decreases the number of small-diameter collagen fibrils relative to the number of greater and stronger collagen type I fibrils, might explain the development and enlargement of posterior staphyloma in pathologic myopia [29].

Likewise, the modifications of the vitreoretinal relationships in high myopia appear to be somehow correlated with a disturbance of collagen metabolism. As previously stated, vitreous liquefaction occurs earlier in myopic eyes and appears to be accompanied by a reduction in the concentration of collagen type IX, causing the collapse of collagen type II, the primary component of the scaffold of the normal vitreous gel [30]. This process results in a significant reduction in the gel volume and a consequent increase in the liquid volume, which leads to posterior vitreous detachment or posterior vitreoschisis. Moreover, in pathologic myopia, posterior vitreous detachment is often complicated by sheets of residual cortical vitreous that remain attached to the inner surface of the retina. These sheets may subsequently contract, contributing to a number of vitreoretinal diseases [31].

Atrophic changes in the retinal pigment epithelium and choroid, lacquer cracks, choroidal neovascularization, peripheral retinal degenerations, foveal RD, macular holes, and foveal retinoschisis are all subsequent complications of pathologic myopia. The etiology of these complications falls into two general biomechanical schemes. The first implicates vitreous traction as the vitreous adheres strongly to the macular area and exerts axial tractional forces that induce the neuroretina to become detached or to split. The second involves tangential stretching forces, created within an enlarging staphyloma, which may induce a stretching mechanism. Chorioretinal atrophy may alter the pumping efficiency of the retinal pigment epithelium, thus influencing the strength of adhesion and leading to further weakening of mechanical attachments and the promotion of RD [32–35].

## 3. Current Trends in the Treatment of Vitreoretinal Diseases in Myopic Eyes

The prognosis of RD in highly myopic eyes has long been considered very poor. The challenge arises from the diversity of lesions at the origin of the RD. These lesions can be peripheral retinal tears, posterior paravascular tears, and macular holes. Moreover, the vitreous base and consequently the peripheral tears are more posterior in highly myopic eyes. These features make the location of the indentation more difficult and hazardous. The presence of chorioretinal atrophy precludes laser treatment, leading to poor retinal adhesion to the underlying pigment epithelium.

There are two methods for closing retinal breaks when repairing RD: external tamponade, using a scleral buckling procedure, or internal tamponade after a pars plana vitrectomy, using the surface energies of intraocular agents introduced at the end of the procedure. Sometimes both are convenient. Scleral buckling may be localized to a limited portion of the eye wall, involving the support of the retinal breaks (plombage), or it may be spread over the whole circumference of the eye in the case of multiple or giant breaks (known as an encircling band, cerclage, or 360° buckling).

Pars plana vitrectomy is increasingly used for primary repair of RD, especially for pseudophakic RD. It involves the removal of the vitreous humor, direct retinal reattachment using a perfluorocarbon liquid, and its substitution with a temporary tamponade agent. After the surgical procedure, the introduction of a tamponade agent prevents seepage of fluid through the causative break, allowing sufficient time for chorioretinal adhesion to occur after laser endophotocoagulation. Internal tamponade agents principally include silicone oil, gas mixtures, and, more recently, HSO.

A longer-acting gas tamponade (C_2_F_6_ or C_3_F_8_) is preferable for RD secondary to breaks in the posterior region (macular holes or paravascular tears) [36]. The aim is to increase the contact time between the macula and the choroid by counteracting the stretching forces caused by the discrepancy between the posterior curvature and the surface of the retina. Laser photocoagulation and cryopexy produce less consistent and permanent adhesions on atrophic and pale areas [36].

Myopic foveoschisis occurs in 8–34% of highly myopic staphylomatous eyes [33, 37] and is mainly characterized by intraretinal cleavage in a myopic posterior staphyloma, often combined with foveal detachment, a lamellar macular hole, ERM, or vitreomacular traction. Many studies have shown that vitrectomy, with or without internal limiting membrane (ILM) peeling and gas tamponade, is an effective treatment for myopic foveoschisis [38–41]. However, Gaucher et al. [42] reported that a macular hole may develop spontaneously or possibly even after vitreous surgery, as observed in nine of the 29 eyes assessed in their long-term follow-up study. In myopic foveoschisis both air and gases appear to be effective tamponades. Hwang et al. [41] reported that the visual acuity of patients with a partial air tamponade did not differ from that of patients with a C_3_F_8_ gas tamponade at the final follow-up. However, this is in contrast with the observations reported in Zheng et al.'s [43] study of the surgical outcomes of vitrectomy for myopic foveoschisis. They found that C_3_F_8_ tamponade resulted in a more rapid anatomical resolution and a greater improvement in visual acuity than a balanced saline solution.

A macular hole is the most common cause of RD in highly myopic eyes [35, 44]. The published literature stresses that myopic eyes with a large posterior staphyloma and an extensive area of atrophy do not respond well to surgical treatment due to the loss of chorioretinal tissue and retinal pigment epithelium atrophy [44, 45]. In the past, a number of studies have indicated that pneumoretinopexy might be a suitable treatment for RD arising from a macular hole in myopic eyes, with a success rate ranging from 54% to 83% [46–48]. However, since the development of pars plana vitrectomy, few authors have recommended pneumoretinopexy as the primary procedure for RD due to a macular hole, as the outcome of pneumoretinopexy is often unpredictable [49, 50]. Notwithstanding this tendency, Kuo et al. [51] strongly recommend gas tamponade as the first-line approach for patients with an RD involving only the posterior staphyloma and suggest that vitrectomy is of potential benefit only for highly myopic patients with poorer visual acuity and a greater extent of RD. Evidence from the literature underlines the importance of posterior vitreous detachment as a key prerequisite for the success of pneumoretinopexy [44, 52, 53].

Treatment of macular holes in highly myopic eyes via pars plana vitrectomy with gas injection (C_3_F_8_ or SF_6_) has been reported to have a primary anatomical success rate ranging from 42.8% to 77.8% [54–57]. However, variations in the number of successful outcomes when vitrectomy is used as the initial procedure in eyes with a large posterior staphyloma and an extensive area of atrophy have led to the introduction of other surgical procedures to improve the success rate. Numerous authors have reported the efficacy of pars plana vitrectomy with peeling of the ERM as a treatment for RD in myopic eyes with macular holes. This approach is based on the hypothesis that tangential traction exerted by the ERM contributes to the development of the macular hole [55, 58–60]. A more sophisticated technique involves a pars plana vitrectomy combined with ILM peeling, and it has a success rate ranging from 69% to 93.8% [58, 61–64].

Silicone oil has also been used for the treatment of RD in myopic eyes with a macular hole [44]. It has several advantages, including a shorter duration of prone positioning and faster recovery with rapid postoperative restoration of visual function. Moreover, the hyperopic shift induced by the silicone oil reduces the myopia, which is appreciated by patients. Silicone oil has a success rate ranging from 66.6% to 100% [53, 54, 65, 66]. High-density silicone oils were compared with standard silicone oil by Avitabile et al. [67] and Mete et al. [68]. The former reported a better anatomical success rate with Densiron than silicone oil in situ, whereas the latter stated that silicone oil and Densiron seemed equally good surgical options since no statistically significant difference in RD was detected.

More recently, macular buckling has been proposed as a first-line treatment for RD in high myopia eyes with a macular hole and posterior staphyloma, because it offers better anatomical results than pars plana vitrectomy [69–72]. This technique is technically challenging due to the difficulties involved in placing the buckling material over the macula. However, it offers the advantage of being able to change the macula from a concave to a convex shape, thereby alleviating the vitreous traction and contributing to connecting the macular hole and the retinal pigment epithelium. Moreover, buckling of the macula leads to a hyperopic shift and a substantial reduction in myopia.

## 4. Physical Properties of Tamponade Agents

An understanding of the physical and chemical properties of intraocular tamponades is paramount for determining their behavior in the eye and for elucidating the means by which they help achieve surgical success. The effectiveness of any internal tamponade is reliant on its ability to establish contact with the internal retinal surface, thereby closing any retinal breaks and holding the neural retina in place against the retinal pigment epithelium. However, some lines of evidence tend to emphasize other properties of the vitreous substitutes, namely, their limitation of the fluid circulation in the vitreous cavity and the reduction of the shear stress on the retinal surface after surgery.

Here, we outline the main characteristics of the endotamponade agents used today in vitreoretinal surgery. Current vitreous substitutes are primarily categorized on the basis of the duration of the tamponade effect, either short term, such as with various gases, or permanent, such as with silicone oil (polydimethylsiloxane, PDMS) and HSO. The latter are water-immiscible and thus permit a long-term tamponade effect. Physical properties that regulate endotamponade behavior include surface tension, the difference between the specific gravity of the agent and the aqueous humor (buoyancy), and viscosity [73].

**Figure 1:**
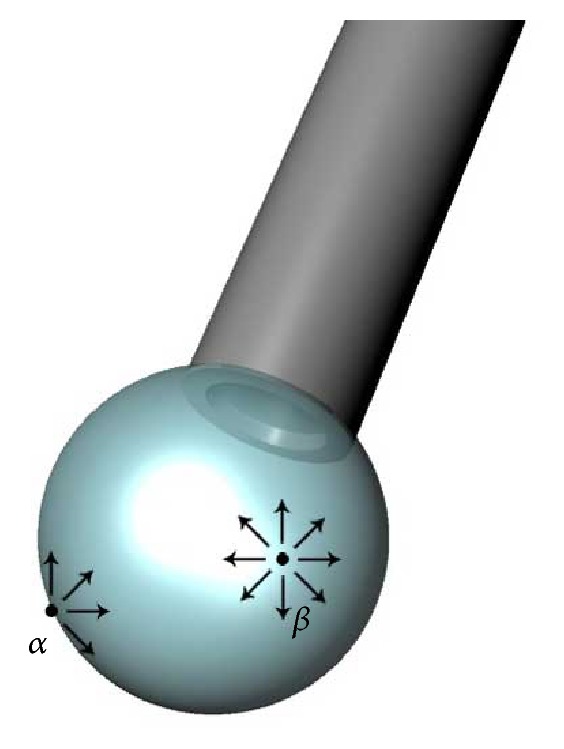
Droplet of water at the end of a pipette. Molecule *β*, which is positioned inside the droplet, is equally attracted in all directions. Molecule *α*, which is located on the surface, is exposed to more attractive forces inside rather than outside the droplet, and the resultant attractive force is thus inwards. Surface tension acts like a film as it tries to achieve the smallest surface area for a given volume (modified from Kirchhof and Wong [74]).

Surface tension is a contractive tendency of the surface of a liquid that allows it to resist an external force; it generally refers to the energy between a liquid and air. Surface tension is also known as “interfacial energy” when it occurs at the interface between two immiscible liquids. The surface tension of the gas or silicone oil is an important factor that prevents the bubble of the tamponade agent from prolapsing through the retinal break, thus facilitating sealing of the break. The physical chemistry of interfaces is complex. Molecules of a liquid are attached to each other by van der Waals forces. In some liquids, such as water, there are also intermolecular polar bonding forces due to an asymmetric distribution of charges between the molecules (Figure 1). On the other hand, in gases, the van der Waals forces are negligible. Thus, at the interface of a fluid and a gas, half of the molecular neighbors within the fluid are missing. The attraction between the neighboring fluid molecules and the fluid molecules along the gas interface generates an inward pulling force on the interfacial molecules, placing the surface under tension. This tension is amplified by the fact that water is a polar substance.

**Figure 2:**
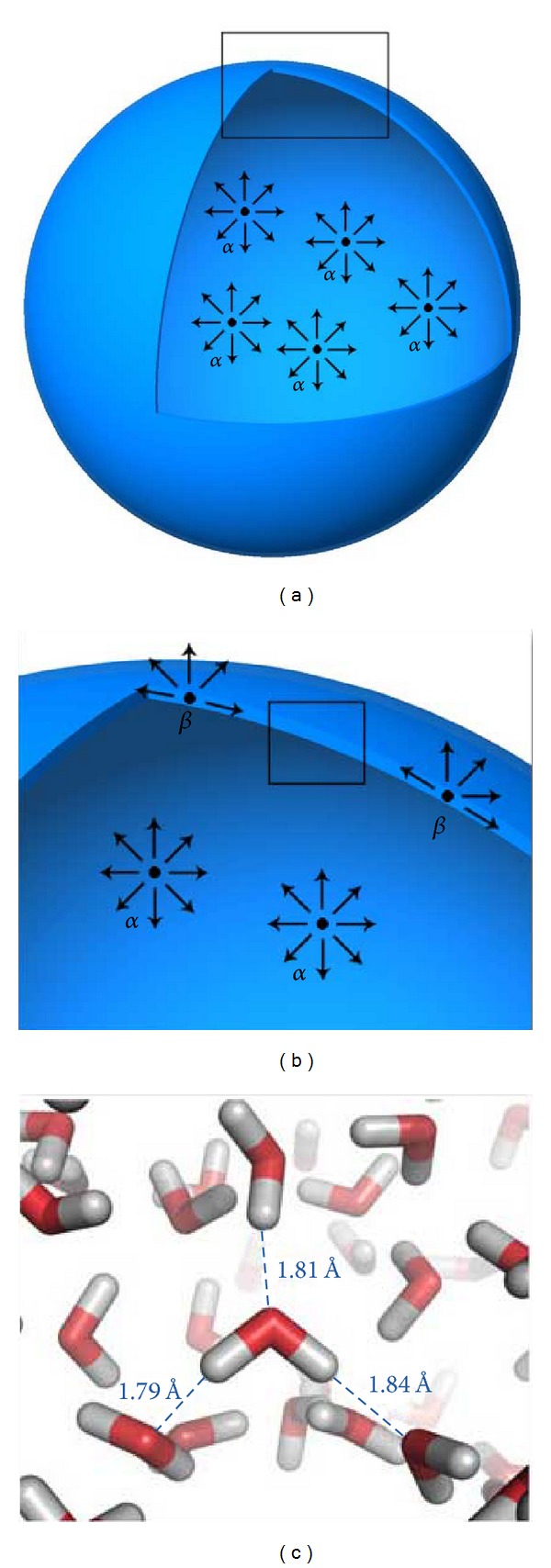
Intermolecular polar bonding forces (c) at the air-water interface are responsible for the surface tension of water. This surface tension makes the water molecules exhibit a skin-like behavior at the surface of the water (b). As a result, when air is forced into water, a spherical bubble is formed (a), which has the least surface area to the highest volume ratio. Molecule *α* = air; molecule *β* = water.

Within the bulk of the liquid, molecules are in constant motion and are continually encountering one another and then moving into another portion of the liquid. This constant motion means that the dipole-dipole forces exerted on each molecule come from all directions, and so, the forces are evened out. However, at the air-water interface of a solution, the intermolecular forces coming from the direction of the air phase are not significant. For this reason, the water molecules at the surface experience a much stronger pull in the side-to-side and downward directions (Figure 2(c)). This has the effect of creating stronger resultant forces (cohesive forces) between water molecules at the surface, namely, the surface tension. Surface tension is what creates skin-like behavior at the surface of water (Figure 2(b)) and is also responsible for the spherical bubble shape of a gas in water (Figure 2(a)). When air (or another gas) is forced into water and bubbles form, surface tension in the thin layer of liquid that forms the skin of the bubbles draws the bubble tightly into the shape, which has the least surface area to the highest volume ratio (which is a sphere), and this is called a minimal surface structure.

When two immiscible liquids are in contact, the molecules of each substance are mostly attracted to similar neighboring molecules. They are also attracted by the molecules of the other fluid, but these attraction forces are weaker. Consequently, a fluid tamponade has less interfacial energy, and the tamponade capacity is less than that of a gas tamponade. For example, air and gases have an interfacial tension of approximately 70 Ergs/cm^2^ at room temperature; perfluorocarbon liquids have an intermediate interfacial tension against water of approximately 50 Ergs/cm^2^, and silicone oil has a relatively low interfacial tension of 36 Ergs/cm^2^ [75].

The specific gravity (the difference between the specific gravity of the agent and that of water) varies depending on the buoyancy in the water. The difference between the specific gravity and the water gravity determines the buoyancy of the agent as well as the shape of the intraocular bubble. The specific gravity of gas is approximately 0.001 g/cm^3^, whereas the specific gravity of silicone oil is 0.97 g/cm^3^ and that of heavy silicone oil is 1.01 g/cm^3^. The specific gravities of the latter are very close to that of water, and, therefore, the buoyancy of silicone oil and heavy silicone oil is low; in contrast, a bubble is made up of gas and has high buoyancy [76]. This results in strong floating of an air bubble in the eye and an upward pressure on the retina, which results in a spherical cap or virtually “D-” shaped bubble with a flat inferior meniscus. This flat side of the bubble contributes to the efficiency of the endotamponade as it increases the area of the bubble that is in touch with the retina. Their greater buoyancy and hydrophilicity also enable gases to easily adapt to the irregular spaces of the eye and to surgical explants if these are present [76]. Consequently, gas and air are the preferred tamponade agents, but they require specific postoperative posturing and are only temporarily present in the vitreous cavity.

On the other hand, a bubble of PDMS has a weak tendency to float and a bubble of HSO has a weak tendency to sink. Thus, both agents have an almost spherical shape in the eye with an evident convex inferior or superior meniscus. Moreover, the overall tamponade efficiency is reduced because part of the volume is used to form the free meniscus instead of establishing contact with the eye cavity. However, this difference might be of little clinical significance if the eye cavity is almost totally filled with the tamponade agent [76].

In contrast, bubbles of perfluorocarbon liquids have an intermediate shape. The bubble is dome-shaped against the retinal surface, but its superior meniscus is flatter than that of PDMS or HSO. The higher specific gravity of perfluorodecalin (1.93 g/cm^3^) and F_6_H_8_ (1.35 g/cm^3^) allows these substances to remain in complete contact with the lower retina. These compounds can flatten the retina owing to their strong sinking force; they fit perfectly against all the irregularities of the posterior pole and the recesses of the indents, and no fluid remains between the inferior retina and the tamponade agent [76, 77].

The weak tendency of a bubble of PDMS to float is not sufficient to overcome its superficial tension, and, thus, a bubble of PDMS cannot fit over steep recesses of the eye wall, such as steep indentations or profound staphylomas. Similarly, a bubble of HSO has a weak tendency to sink and cannot perfectly cover the irregularities of the vitreous cavity.

The behavior of a tamponade agent in the eye is dependent on the interactions among three factors, namely, the aqueous humor, tamponade agent, and retina. For instance, when PDSM is used in the eye cavity, all three are in contact with each other. That is, the retina is in contact with the oil, the oil is in contact with the aqueous humor, and the aqueous humor is in contact with the retina [7]. As previously reported, the shape of an intraocular bubble depends primarily on the specific gravity of the endotamponade agent and then on the interfacial tension with water and hydrophobicity.

The hydrophilic property of the retina keeps it preferentially in contact with the water rather than the agent, contributing to the convex shape of every meniscus. This property also causes a subtle doughnut of fluid to form between the bubble of the endotamponade and the retina. This phenomenon is particularly evident intraoperatively and is sometimes responsible for difficulties encountered during endodrainage of subretinal fluid from peripheral retinal breaks (i.e., it induces slippage), making it difficult to completely remove the water from the eye [74].

Complete drainage of the fluid from the vitreous cavity is practically impossible even after a perfect procedure. Endotamponade underfill may arise either as a result of a poor fluid-air exchange technique or as a result of the compression of the vitreal base remnants by the tamponade intraoperatively or by choroidal decongestion postoperatively. Consequently, the eye is incompletely filled with the tamponade agent, which is thus only partially in contact with the retina (superiorly or inferiorly depending on the specific gravity of the agent).

The viscosity of the material is another crucial factor in maintaining the integrity of the tamponade and in reducing long-term dispersion. Viscosity is the main factor influencing emulsification: the lower the viscosity, the less the mechanical energy needed to disperse a large bubble into small droplets. Intraocular tamponades need to be highly viscous to decrease their tendency to emulsify and disperse into small bubbles that can cross retinal breaks or the zonula and reach the anterior segment, causing inflammation or glaucoma [78, 79]. PDMS with a high viscosity (5000 mPas) is more stable and tends to show less dispersion. It is associated with fewer complications than a less viscous PDMS (1000 mPas) [80]. However, in clinical practice, PDMS is usually removed within 3-4 months, and the dispersion difference may not be significant for this period. The high viscosity of a 5000-mPas PDMS increases the difficulties associated with the handling of the substance. A 1000-mPas PDMS can be introduced and removed much more easily than a 5000-mPas PDMS, and it is thus the agent most commonly utilized by many vitreoretinal ophthalmologists. Moreover, with the advent of minimally invasive surgery (23–25 gauge), it is preferable to use a less viscous silicone oil to save time during its introduction and passage through the small gauge system. An HSO with a low viscosity, which does not induce emulsification, is desirable.

Heavy tamponades have a lower viscosity than PDMS: F_6_H_8_ and the other semifluorinated alkanes (SFAs) have a viscosity of 2.5–3 mPas, which is close to that of water (1 mPas). They are easily handled but tend to emulsify very early after surgery. The dispersion rate of F_6_H_8_ ranges from 30 to 100% after a few weeks, depending on the removal time. Mixing an SFA with a PDMS with a viscosity of 1000 mPas can inhibit the dispersion of F_6_H_8_, but the SFA agent can be unstable, and its stability will vary in response to temperature and eye movements [81, 82].

The mixture of a SFA and a PDMS with a viscosity greater than 5000 mPas results in an HSO compound, which is more stable. These compounds have a higher viscosity than pure SFA: approximately 1400 mPas for Densiron 68 and approximately 3800 mPas for Oxane HD. This property decreases the emulsification rate, but it also increases the handling difficulties.

The extent of emulsification of heavy tamponades is time dependent. Thus, emulsification tendency is the main factor influencing the time of removal of such tamponades. It is necessary to stabilize the retina for a period long enough for proliferative vitreoretinopathy (PVR) to develop (usually 4–6 weeks). The tolerability of new HSO agents has been improved, and this permits these substances to be left in the eye for up to 3-4 months without any detrimental effects [83].

In very few cases, silicone oil endotamponade can lead to an increase in the intraocular pressure (IOP) after vitrectomy [84]. This is likely due to emulsified oil reaching the anterior segment, since no significant difference in angle width before and after vitrectomy has been documented [85]. IOP increase can usually be well controlled by topical antiglaucoma medication and is reversible in most patients after oil removal [84].

According to Boyle's law, gas-filled eyes, after vitrectomy, are affected by variations in atmospheric pressure because of both altitude and weather conditions. Caution should be exercised with regard to altitude changes in patients with intravitreal gas. However, increase in IOP can occur even if there is no increase in altitude [86]. In fact, sudden lowering in atmospheric pressure of approximately 20 hPa (15 mmHg) would result in an IOP increase of 15 mmHg, which would be clinically relevant [87].

Pupillary block is another potential complication after vitrectomy with gas or silicone oil endotamponade in aphakic eyes. The risk of pseudophakic pupillary block after vitrectomy with air tamponade may be increased with inflammation due to combined vitrectomy and cataract extraction as well as zonular weakness, thus allowing air to escape from the vitreous cavity to the anterior chamber [88].

## 5. The Effect of Scleral Buckling on Intraocular Tamponades

There is an ongoing debate on whether vitrectomy or scleral buckling is best for the treatment of RD. Each procedure involves different mechanical effects for reducing the mechanical stress induced by fluid movements and traction on the retina.

From a clinical point of view, retinal breaks located in the inferior quadrants present a surgical challenge because standard intraocular tamponades cannot provide enough direct support to completely cover the quadrants of the inferior retina.

It is virtually impossible to achieve a complete tamponade of the spherical shape of the vitreal cavity using any currently available intraocular agent [7]. Posturing may be beneficial for maintaining contact between the tamponade and retinal tears and for reducing intraocular fluid movement. However, perfect compliance of the patient is difficult to achieve in most cases. Therefore, some surgeons prefer to use apposition of an equatorial encircling scleral buckle combined with vitrectomy and silicone oil (1000–5000 mPas) to increase the endotamponading effect and to improve the chances of closing inferior breaks. The position of the scleral encircling buckle at the equator of the globe changes the shape of the vitreous chamber, reducing the effect of the stressing forces exerted by the vitreous on the anterior retina.

The mere presence of a lens indentation in the vitreous chamber has been shown to change rotational fluid movements, creating additional vortices that might increase the shear stress near the ora serrata, which is the more vulnerable retinal zone [28].

The endovitreal fluid movement is interrupted by the presence of the encircling buckle, which reduces the stress distribution on the anterior retina [89]. Moreover, the scleral buckle changes the subretinal pressure from negative to positive and modifies the intraocular fluid dynamics, causing the fluid to flow out of the subretinal space. Recent studies have shown that the subretinal fluid flows much faster in the area of the “buckle” during eye movement, facilitating outflow from the retinal break since fluid pressure is inversely correlated with fluid velocity (Bernoulli's principle) [90]. Indeed, the subretinal pressure over the buckle decreases during an eye movement that produces a higher fluid velocity. This phenomenon induces a pressure gradient that pushes the retina against the back of the eye, facilitating resolution of the RD. These considerations are contradictory to the conventional wisdom of attempting to minimize eye movements. Rapid eye movements are today expected to facilitate more rapid retinal reattachment and to improve outflow of subretinal fluid.

The presence of an indentation stabilizes the intraocular silicone bubble during eye movements by reducing its rotation as well as the shear stress induced by the tamponade agent on the retina. The reduced shearing forces stabilize the retinal breaks and reduce silicone oil emulsification [91].

Care should be taken to support a retinal break when using scleral buckling to ensure maximal filling and accurate mounting of the buckle where the break is positioned at the apex of the indent in the oil bubble [76]. The use of scleral buckling may enhance the overall internal tamponade effect of an oil bubble in parts of the retina away from the site of the indent. For example, the use of a 360° buckle placed at the equator of the eye wall may enhance the oil tamponade effect on a posterior retinal break or an inferior retinotomy edge [76]. A light indentation with a subtle encircling band or a gentle broad buckle is suggested when silicone oil is used to avoid the formation of sequestered fluid in front of and behind the buckle.

Some surgeons use a subtle encircling buckle (e.g., 240 style, 2.5 mm) that is not markedly tightened, while other authors have suggested the use of a larger buckle (e.g., 287 style, 7 mm) because it creates a more posterior ora serrata that reduces the traction of incompletely removed ERMs and avoids the complications associated with thinner buckles [7, 92]. This technique is considered effective, although it noticeably prolongs the surgical time and thus increases the surgical risks [93].

However, a few studies have shown that vitrectomy with gas, air, or PDMS tamponade without the application of a scleral buckle may also be safely used for treating inferior break RD in the absence of proliferative vitreoretinopathy (PVR); furthermore, it can achieve results comparable to vitrectomy combined with the use of a scleral buckle [94–97]. It is known that silicone oil can support the superior and inferior fundi simultaneously without an encircling buckle, as is the case in macular translocation with a 360° retinotomy [98, 99].

Several studies have shown that vitrectomy with gas alone achieves comparable anatomic outcomes compared with vitrectomy with a scleral buckle in eyes without or with PVR. Visual acuity is reportedly more significantly improved when a vitrectomy is performed and no scleral buckle is used [96, 100–102]. Heavy silicone oils (Densiron 68 and Oxane HD) have also been shown to be effective for treating rhegmatogenous RD with inferior breaks and PVR without scleral buckling [103–105].

Despite these reports, there is no conclusive evidence to date as to whether buckled or nonbuckled vitrectomy is preferred for use with classical endotamponade agents for repairing inferior break RD with PVR during routine surgery.

## 6. Effects of Tamponade on Retinal Function

The development of a vitreous substitute that can mimic natural vitreous tamponading all over the retinal surface and that can also be left in place is both a challenging and an attractive field of research in ophthalmology.

Clinical and experimental evidence has shown that agents that exert an optimal endotamponade are very effective anatomically but are not biocompatible. In this respect, intraocular gases fit every irregularity of the vitreous cavity, but they may also cause visual field defects [106–109]. Desiccation of the retina during fluid-air exchange with air seems the most likely mechanism responsible for acute loss of the peripheral visual field that occurs in some vitreoretinal procedures, and it may be prevented only by incomplete filling or if the gas is humidified before its infusion into the eye [110, 111].

The high specific gravity of perfluorodecalin (1.93 g/cm^3^) and F_6_H_8_ (1.35 g/cm^3^) allows these substances to remain perfectly in contact with the inferior retina. These compounds can flatten the retina by means of their strong sinking force; they fit perfectly over all the irregularities of the posterior pole and the recesses of the indents, without any fluid being retained between the inferior retina and the tamponade agent. However, the specific gravity of these agents is probably too high, and the absence of water between the agent and the inferior retina produces a mechanical or metabolically negative effect that impairs retinal function [112].

Retinal alterations such as outer and inner photoreceptor damage, narrowing of the outer plexiform layer, ultrastructural distortions of photoreceptor outer segments, migration of the receptor cell nuclei to the photoreceptor layer, and inflammatory responses due to ischemic effects have been reported following the use of heavy liquids such as perfluorocarbon, fluorosilicone, and F_6_H_8_ [113, 114]. Experimental studies confirmed pathologic vascular and structural changes of the retina after the use of heavy endotamponade agents. A few weeks of endotamponade with perfluorocarbon (PFC) appeared to cause ultrastructural changes in the inferior retina of rabbits [115–117].

Mechanical pressure on the retina may be partly responsible for the changes observed in the inferior retina when heavy agents are used. These changes may represent a mechanical rather than a toxic effect as similar changes have been reported in the superior retina in silicone-filled eyes. The specific gravity of PFC ranges between 1.7 g/cm^3^ and more than 2.0 g/cm^3^. In part, at least, the histologic changes in the retina can be attributed to the dystrophic effect of the “heavy” liquids pressing on the inferior retina. However, retinal damage has been shown to be more evident in the external layers rather than the inner retinal layers that are in direct contact with the heavy substance. Moreover, a simple calculation of the increase in pressure over the inferior retina has shown this to be minimal (<0.6 mmHg in an eye with a diameter of 2.2 cm) and within the normal diurnal range of pressure changes in the eye [77]. The mechanism responsible for the damage is thus considered different from a simple mechanical interaction.

Gravity might not be causally linked to retinal damage, which might rather be the result of a metabolic disturbance. Indeed, recent observations appear to indicate that PFC toxicity is not primarily due to the high specific gravity or possible chemical impurities but rather due to the inability to dissolve ions. Optical coherence tomography measurements indicate that PFCs, including the low specific gravity semifluorocarbon, replace most of the aqueous sink volume available for potassium siphoning. Impairment of retinal K^+^ clearance may be an important mechanism of PFC-induced retinal injury [112]. These observations provide a better explanation for the morphological alterations reported in Müller cells, leading to the subsequent atrophy of the photoreceptors that occupy the external retinal layers. These cells have been shown to develop features of reactive gliosis, including hypertrophy, expression of glial fibrillary acidic protein, and drop-like protrusions between the inner segments of the photoreceptors. The “heavier” heavy tamponade agents are hydrophobic and exclude the aqueous humor from the surface of the retina. A fine film of water is necessary for the Müller cells to function and for the diffusion of metabolites and oxygen to the inner retina [118].

The presence of a hydrophobic and light endotamponade agent such as PDMS leads to poor contact with the retina, permitting oxygenation and ionic flux across the posterior pole due to the thin layer of water present around the tamponade bubble. Moreover, vitrectomy has been shown to increase oxygen diffusion from the anterior region to the posterior segment of the vitreous cavity [119, 120]. Some ischemic retinal diseases may therefore be treated and partially ameliorated after complete elimination of the vitreal cortex and the premacular posterior hyaloid because of the increase in oxygen diffusion. Besides, iris rubeosis or neovascularization due to retinal ischemia may be reduced after the introduction of PDMS in diabetic patients [121, 122].

On the other hand, silicone compartmentalizes the eye and confines angiogenic substances to the posterior segment, causing entrapment of retina-derived angiogenic substances between the retinal surface and the silicone oil bubble, which leads to perisilicone proliferation or PVR [123]. Such compartmentalization was confirmed by de Juan E Jr. et al. [124], who reported that PDMS prevents the decrease in anterior chamber oxygen tension that occurs after lensectomy and vitrectomy in cats and humans.

“Lighter” heavy tamponades with a lower specific gravity, such as Oxane HD and Densiron 68 (specific gravity 1.02 g/cm^3^ and 1.06 g/cm^3^, resp.), are less toxic than PFC and fluorosilicone, despite a reduction in their tamponade effect, especially in the presence of retinal indents [125].

HSO offers some advantages in managing inferior breaks, although the tamponade effect of HSO has not been shown to be either inferior or superior to that of PDMS in almost all clinical series. In fact, both behave as silicone oils, but on opposite sides. The rate of PVR in HSO-treated patients is similar to that in PDMS-treated patients, but HSO tends to shift the site of PVR to the upper retina above the horizontal meridian. The presence of a subtle meniscus of fluid around bubbles with a specific gravity very close to that of water is probably the main reason for the diffusion of growth factors and cytokines from the inferior breaks to the upper retina, which results in epiretinal proliferation [103].

## 7. Discussion

The management of retinal detachment in a high myopic eye, with posterior staphyloma, poses a challenge for the retinal surgeon. The conventional rationale for performing a vitrectomy is to relieve vitreoretinal traction and obtain a temporary endotamponade effect in order to seal retinal breaks and to reposition the detached retina against the retinal pigment epithelium. This blockage has to be maintained unless a permanent chorioretinal adhesion forms in response to cryotherapy or laser photocoagulation. Sealing is particularly important in the myopic eye since the presence of chorioretinal atrophy delays the adhesion and often precludes laser treatments.

The use of silicone oil tamponades in the treatment of complex RDs is well documented and has been an accepted clinical practice since the 1970s. From a materials science point of view, the important properties of silicone oils that enable the sealing effect are their interfacial tension with the remaining aqueous humor, viscosity, and specific gravity [126]. As previously reported, both gas and air have an optimal barrier function in the filled area, while PDMS is much weaker in this respect. In particular, the low specific gravity of PDMS and gas leaves the inferior retina exposed and also potentially bathed in inflammatory mediators [127]. Therefore, the tamponading theory does not fully explain their ability to repair an RD with inferior tears, where no direct contact between the tamponade and the retina is possible. Furthermore, agents with a poor inferior endotamponade capacity can seal a 360° retinotomy, as in macular translocation, or seal macular holes without complete tamponade of the central fovea [128]. It is therefore evident that other factors come into play in addition to the sealing effect of the tamponades.

Both saccadic eye movements and head movements generate forces in the liquefied vitreous humor that can rip and detach the retina. In a similar manner, these movements create a reduced but continuous shear stress in a vitrectomized eye, which, in some cases, might overcome the adhesive force between the retina and the retinal pigment epithelium, leading to recurrent RD. As previously reported, the traction caused by vitreous movements on the inner retina increases along with the eyeball diameter and is thus significantly higher in myopic individuals. In high myopia (−20 D), the maximum wall shear stress can be more than 1.5 times stronger than that in emmetropic eyes [21, 129].

**Figure 3:**
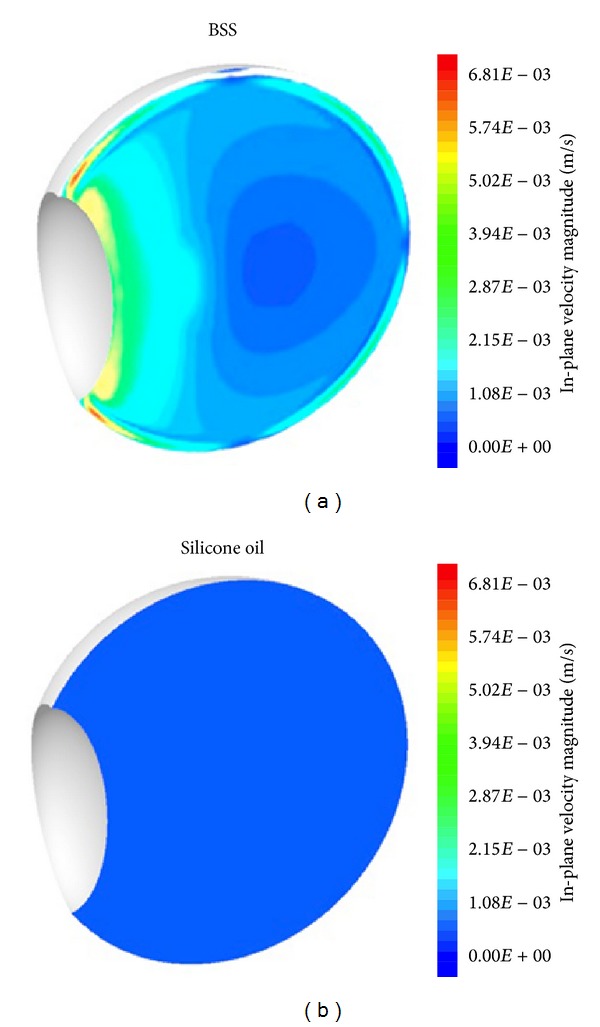
In-plane velocity magnitude contours on the vertical plane containing the rotational axis for BSS and silicone oil (modified from Abouali et al. [129]).

Abouali et al. [129] modeled eye motion using a dynamic mesh technique and assessed the fluid dynamics in the vitreous cavity due to saccadic eye movement. They compared PDMS with glycerol (as did Repetto et al. [130] and Stocchino et al. [131]) and reported that PDMS leads to a lower shear stress on the walls despite its higher viscosity. This is probably due to the lower density of PDMS, which dampens the saccade movements. However, in their model, the shear stress on the retina from silicone oil arising from saccade movements was nearly 20 times that of the liquefied vitreous humor or balanced salt solution (BSS), because silicone oil has a much higher viscosity, which ensures that it flows in a similar manner to the bodily rotation. Nevertheless, in the case of water, the velocity gradient normal to the wall was much higher. This is probably the reason for the increased risk of recurrent RD in postvitrectomy eyes refilled with BSS. Moreover, the in-plane velocity magnitude in the vertical plane containing the rotational axis was reported to be higher for the liquefied vitreous and BSS. This flow has a lower intensity for the silicone oil, after a 50° saccade (one-fifth compared with that of BSS; Figure 3) [129]. In highly myopic eyes treated with silicone oil as endotamponade, there should be no theoretical difference whether standard or heavy silicone oil is used, and whether the patient maintains a strict head posturing after the surgery, because it is a matter of low shear stress that closes the macular hole.

In contrast, low fluid shear forces on the retinal wall after vitrectomy and gas tamponade are generated by both saccadic eye movements and head movements. The gas-filled area experiences shear stresses that are two to three orders of magnitude less than those in the fluid-filled area and are hence negligible, as found by Angunawela et al. [132] after vitrectomy in a model eye. However, it could be too simplistic to consider fluid shear stress in a model eye as the only factor relevant to retinal attachment after vitrectomy. Indeed, the residual vitreous is often neglected and a complete vitrectomy is necessarily assumed at the time of surgery for the purpose of computational modeling.

In the highly myopic eye, the vitreous cortex plays a fundamental role. An anomalous posterior vitreous detachment is frequent and occurs when the extent of vitreous liquefaction exceeds the degree of weakening of the adhesion at the vitreoretinal interface. In the myopic eye, often, the vitreous cortex remains attached to the retina despite the apparent posterior vitreous detachment, as appreciable with a Weiss ring. This situation leads to vitreoschisis, where the increasing stiffness of the preretinal vitreous is thought to be the cause of abnormal tangential traction at the vitreoretinal interface either in the middle periphery or at the posterior pole, thereby causing macular pucker, macular hole, and myopic traction maculopathy [133].

The role of endotamponades in myopia is crucial. Heavy tamponades are an attractive option for tamponading inferior retinal tears. However, they have a specific gravity that is only slightly higher than 1 (1.06 g/mL for Densiron 68 compared with 1.02 g/mL for Oxane HD); therefore, although they sink, they also have a near-spherical shape, and consequently, their tamponade efficiency is low. To further increase the specific gravity and thus increase the difference with respect to the aqueous humor, silica particles can be added to the tamponade fluid. Williams et al. [126] recently tested this theory by using a rabbit model. They found that this alteration significantly changed the shape of the bubble compared with standard HSO, providing an opportunity to tailor the specific gravity by varying the amount of material added. The optical clarity of the tamponade was maintained by matching the refractive index of the silica and the silicone oil.

In conclusion, gas and air have the best properties for use as tamponade agents, but their effects are temporary and are mostly limited to the upper retina; silicone oil and HSO do not provide as good a tamponade effect, but these are also efficacious as they modify the retinal shear stress induced by the fluid as well as the fluid dynamics in the vitreous cavity. In a number of situations, such as myopic macular holes with or without RD, myopic foveoschisis, penetrating ocular injuries with RD, and inferior giant retinal tears, treatment with HSO might be considered. The advantages of using HSO instead of PDMS include decreased surgical time and easier handling.

Anatomic and visual outcomes following surgery are often limited in myopic eyes because of their specific anatomy, and determining the appropriate use of each vitreous substitute may be challenging. A longer-action endotamponade in highly myopic eyes might be considered to increase retinal reattachment rate and final visual outcome, especially in RD associated with macular holes. A shorter-acting gas might not provide a long-enough tamponade effect to allow for a glial reaction responsible for the closure of the macular hole and posterior retinal reattachment. This is especially relevant in highly myopic eyes, where the chorioretinal adhesion may not be as firm as it would be in patients with a healthy retinal pigment epithelium [134].

The recurrence rates for complicated RD are as high as 20–25%, and although vitreoretinal techniques have been improved in recent years, especially with the advent of HSOs, the rate of PVR has not significantly decreased [103, 135]. On the other hand, as previously reported, the subtle meniscus of fluid around the silicone oil allows diffusion of growth factors and cytokines from the retinal tears and consequent epiretinal proliferation.

The vitreous substitutes available to date do not provide a perfect endotamponade effect due to the physical characteristics of the available agents or the risk of damaging the retinal physiology, especially in myopic eyes. The natural vitreous body acts as a viscoelastic damper; it is hydrophilic, permits diffusion of oxygen, ions, and small particles, and blocks fluid movement and shear stress over the retina. Therefore, an ideal vitreous substitute for both myopic and emmetropic eyes should mimic such properties and be easy to manage. Many substances with similar characteristics have been studied, such as polyvinyl alcohol hydrogel [136], cross-linked biopolymer hyaluronic acid [137], and thermosetting gel [138]. However, several difficulties were encountered at the time of injecting these substances through small gauges inside the eye, and thus far, no vitreous substitute has been shown to remain unchanged and maintain its own physical characteristics in vivo.
